# Supplementary material for: Homozygous YME1L1 mutation causes mitochondriopathy with optic atrophy and mitochondrial network fragmentation
Source: eLife. 2016 Aug 6;5:e16078. doi: 10.7554/eLife.16078 (PMC4991934; doi:10.7554/eLife.16078)
Supplement: Supplementary file 1. — (A) List of primers for qPCR and genotyping. (B) List of primary and secondary antibodies. DOI: http://dx.doi.org/10.7554/eLife.16078.019 [file elife-16078-supp1.docx]

**Supplementary file 1A: List of primers for qPCR and genotyping**

| **Primer/Probe** | **Species** | **Sequence 5´-3´** |
| --- | --- | --- |
| *YME1L1* mRNA expression | | |
| mYme1l1-F | mouse | AGGCGAACTCGTCTGATCCTC |
| mYme1l1-R | mouse | ACAGGGTCTACCGCAGAATCAA |
| mYme1l1-probe | mouse | FAM-TCCTGTAGTTGTCCGAAAGCGCACA-BBQ |
| mHprt-F | mouse | ATCATTATGCCGAGGATTTGGAA |
| mHprt-R | mouse | TTGAGCACACAGAGGGCCA |
| mHprt-probe | mouse | FAM-TGGACAGGACTGAAAGACTTGCTCGAGATG-TAMRA |
| hYME1L1-F | human | CCCATGTCTCTGCACAATCC |
| hYME1L1-R | human | ACCCCTTCACGAATGATGG |
| hYME1L1 probe | human | FAM-CAGCTAATCTCTCGGAGGTAGACTGGAGAC-BBQ |
| hRPII-F | human | GCACCACGTCCAATGACAT |
| hRPII-R | human | GTGCGGCTGCTTCCATAA |
| hRPII-probe | human | FAM-TACCACGTCATCTCCTTTGATGGCTCCTAT-TAMRA |
| *YME1L1* genotyping | | |
| YME1L1-Genotyping-F | human | CGTATTGAAGTGTCAGGCAAA |
| YME1L1-Genotyping-R | human | TCAGCCAACAAAAGAAATCAGA |
| *YME1L1* Sanger sequencing of putative patients | | |
| YME1L1-Sanger- Exon5-F | human | TCTTAGGCATTTTTCTTTTCCTG |
| YME1L1-Sanger-Exon5-R | human | TGAAAATATTTGCTTTTTGGATCA |

**Abbreviations**: mYme1l1, mouse Yme1l1; hYME1L1, human YME1L1; mHprt, mouse Hprt; hRPII, human RPII; YME1L1-Sanger-Exon5, YME1L1 Sanger sequencing for Exon 5.

**Supplementary file 1B: List of primary and secondary antibodies.**

| **Name** | **Source** | **Catalog No.** | **Host** | **Dilution** |
| --- | --- | --- | --- | --- |
| ATP-5B | Thermo Fisher Scientific | A-21351 | mouse | 1:200 |
| COX4 | Cell signaling | 4844 | rabbit | 1:1000 |
| CYTOCHROME c | BD Pharmingen | 556432 | mouse | 1:1000 |
| CYCLOPHILIN D | Abcam | ab110324 | mouse | 1:1000 |
| NDUFB6 | Abcam | ab110244 | mouse | 1:1000 |
| OPA1 | BD Transduction Lab. | 612607 | mouse | 1:1000 |
| PRELID1 | Abnova | H00027166-M01 | mouse | 1:500 |
| PD41 | Cell Signaling | 3936 | Mouse | 1:1000 |
| TOMM20 | BD Transduction Lab. | 612278 | mouse | 1:1000 |
| TOMM20 | Santa Cruz | sc-11415 | rabbit | 1:1000 |
| SDHA | Abcam | ab14715 | mouse | 1:10000 |
| YME1L1 | Proteintech | 11510-1-AP | rabbit | 1:1000  1:100 (IC) |
| anti-mouse HRP | Dako | P0447 | goat | 1:10000 |
| anti-mouse Cy3 | Invitrogen | A10521 | donkey | 1:1000 |
| anti-mouse 488 | Invitrogen | A21121 | goat | 1:1000 |
| anti-rabbit HRP | Amersham Biosciences | NA934 | donkey | 1:2000 |
| anti-rabbit Cy3 | Invitrogen | A10520 | donkey | 1:1000 |
| anti-rabbit Alexa 488 and anti-mouse Alexa 568 | Invitrogen | A-11008 and A11004 | goat | 1:500 |

**Abbreviations:** ATP Synthase Subunit beta, ATP-5B; Cytochrome c oxidase subunit 4, COX4; NADH ubiquinone oxidoreductase 1 beta subcomplex 6, NDUFB6; optic atrophy 1, OPA1; PRELI domain containing protein 1, PRELID1; translocase of outer membrane 20, TOMM20; succinate dehydrogenase, SDHA; yeast mitochondrial escape 1 like protein 1, YME1L1; Immunocytochemistry, IC.
